# A novel social distance model reveals the sidewall effect at bottlenecks

**DOI:** 10.1038/s41598-021-00486-1

**Published:** 2021-10-25

**Authors:** Xinyu Si, Lei Fang

**Affiliations:** Department of Civil and Environmental Engineering, University of Pittsburgh, Pittsburgh, PA 15260 USA

**Keywords:** Statistical physics, Nonlinear phenomena

## Abstract

Intermittent and periodic outbreaks of infectious diseases have had profound and lasting effects on societies throughout human history. During the global spread of SARS-CoV-2 and the resulting coronavirus disease (COVID-19), social distance has been imposed worldwide to limit the spread of the virus. An additional deliberate intention of keeping a minimum safety distance from neighbors can fundamentally alter the “social force” between individuals. Here, we introduce a new “social distance” term inspired by gas molecular dynamics and integrate it into an existing agent-based social force model to describe the dynamics of crowds under social-distanced conditions. The advantage of this “social distance” term over the simple increasing of the repulsive range of other alternatives is that the fundamental crowd properties are precisely described by our model parameters. We compare the new model with the Helbing and Molnar’s classical model and experimental data, and show that this new model is superior in reproducing experimental data. We demonstrate the usability of this model with a bottleneck motion base case. The new model shows that the bottleneck effect can be significantly alleviated through small wall modifications. Lastly, we explain the mechanism of this improvement and conclude that this improvement is due to spatial asymmetry.

## Introduction

Modeling a social-distanced crowd is of great importance because social distancing is one of the promising ways of reducing disease transmission during pandemic, post-pandemic, and flu season. Within a social-distanced crowd, individuals move and maneuver with an additional deliberate intention of keeping a minimum safety distance from their neighbors, which can lead to fundamentally unique behaviors. Moreover, the extra “buffer” space around the individuals imposes a significant constrain on crowd moving efficiency. We are facing a situation where we do not have means that would help us to assess, estimate, and model operations of walking crowds in the new reality of corona-impacted conditions.

Crowd dynamics simulation has a long history^1^. There are approaches over different scales: Micro-scale^2^, Meso-scale^3^ and Macro-scale^4,5^. One of the most widely accepted crowd dynamics models is the agent-based social force model^2,6–8^. The concept of “social force” is introduced to simulate the human crowd, which states that any individual’s velocity change is equal to the sum of the forces (per unit mass) on that individual. This kind of simulation turns out to be an effective way of reproducing empirical crowd flow observations robustly, especially when such flows are considered from a statistical perspective^1^. This may due to the fact that humans in a crowd react subconsciously rather than making complicated decisions and, hence, follow a set of simple (yet unknown) reaction rules^1^.

The traditional agent-based social force model cannot be directly used to learn the dynamics of crowds under this special social-distanced period. Here, we report a novel “social distance” term, which is integrated into an existing agent-based social force model^1,2,9^ to simulate the crowd with various imposed social distances. We will compare the new model with Helbing and Molnar’s classical model^2^ and also compare the model results with that from experiments. With this model, we want to answer the question: can small modifications to existing infrastructures increase transportation efficiency in the pandemic and post-pandemic period, where social distances being imposed? In order to fully optimize the transportation efficiency of the existing infrastructure, there are several motion base cases to study^10^. Here, we focus on the most common limiting case: bottleneck flows at doors. In this paper, we first introduce the agent-based model with an emphasis on the new “social distance” term in “Social distance model” section. Second, we compare the new model with Helbing and Molnar’s classical model and compare the results from both model with experimental results in “Results” section. In “Discussions” section, we demonstrate the effects of social distancing on the human flow rate through bottlenecks. Then, we show that by introducing asymmetry near the bottlenecks, we can significantly increase the transportation efficiency of the bottlenecks. Lastly, we conclude our paper with “Conclusions” section. This paper demonstrates the new possibility of the adapted agent-based crowd model in optimizing transportation efficiency in pandemic and post-pandemic times, and we conclude our paper by identifying opportunities for future works.

## Social distance model

We followed the agent-based social force model framework, which has been explained in detail elsewhere^2,9,10^. Thus, we introduce this model only briefly while focusing on the new term developed to describe the social distancing effect during the pandemic and post-pandemic periods. The model assumes that there are *N* individuals *i* in the crowd with locations ${\mathbf{r}}_i(t)$ and velocities ${\mathbf{v}}_i(t)$. Each individual also has a desired speed $v_i^D$ and is limited by a maximum speed $v_i^{max}$. The change of an individual’s speed is interpreted as the consequence of the summation of three major “social forces” on the individual.

First, the individual intends to reach a destination (${\mathbf{r}}_i^{D}$) as directly as possible with the desired speed $v_i^D$. This leads to the desired direction

1 $$\begin{aligned} {\mathbf{e}}_i(t) = \frac{{\mathbf{r}}^{D}_i - {\mathbf{r}}_i(t)}{ \left\Vert {\mathbf{r}}^{D}_i - {\mathbf{r}}_i(t)\right\Vert }. \end{aligned}$$

Therefore, the adaptation of the individual’s velocity from $\mathbf{v_i}(t)$ to $v_i^D {\mathbf{e}}_i(t)$ with a time scale $\tau _i$ can be described as an acceleration term:

2 $$\begin{aligned} {\mathbf{F}}^{D}_i = \frac{v_i^D {\mathbf{e}}_i(t) - {\mathbf{v}}_i(t)}{\tau _i}. \end{aligned}$$

The second term considers the fact that an individual will tend to keep a certain distance from walls^2^. Here, we only consider the effect of the closest wall on the individual. The acceleration of individual *i* to the wall *W* is described as:

3 $$\begin{aligned} {\mathbf{F}}_{iW}({\mathbf{r}}_{iW}) = -c_{w} \nabla _{{\mathbf{r}}_{iW}} U_{iW}( \left\Vert {\mathbf{r}}_{iW}\right\Vert ), \end{aligned}$$

where $c_{w}$ is a direction dependent coefficient that accounts for the sight effect of the individual. The weight $c_{w}$ can be lower for the case where the closest point of the wall is outside the sight of the individual^2^. Besides, $U_{iW}$ is a repulsive monotonic decreasing potential and ${\mathbf{r}}_{iW} = {\mathbf{r}}_{i} - {\mathbf{r}}_{W}$ with ${\mathbf{r}}_{W}$ defined as the closest point on the wall to the individual. $\nabla _{{\mathbf{r}}_{iW}}$ is the directional derivative along ${\mathbf{r}}_{iW}$. For the repulsive potential $U_{iW}$, we followed the Helbing and Molnar’s form^2^: $U_{iW}( \left\Vert {\mathbf{r}}_{iW}\right\Vert ) = U_{iW}^0 e^{-\left\Vert {\mathbf{r}}_{iW}\right\Vert /R}$, where $U_{iW}^0$ and R are constants. The derivative of $U_{iW}$ along ${\mathbf{r}}_{iW}$ gives the repulsive force (per unit mass) between *i* and *W*, which monotonically decreases with increasing distance between *i* and *W*.

The third term, which is the new term that we introduce, describes the forces between individuals when social distance is imposed. Inspired by the gas molecular dynamics^11^, we describe the repulsive potential $V_{ij}$ between individual *i* and *j* as a generalized form of Lennard-Jones potential function:

4 $$\begin{aligned} V_{ij}(\left\Vert {\mathbf{r}}_{ij}\right\Vert ) = \epsilon ((\frac{\sigma }{\left\Vert {\mathbf{r}}_{ij}\right\Vert })^{2n} - (\frac{\sigma }{\left\Vert {\mathbf{r}}_{ij}\right\Vert })^{n}), \end{aligned}$$

where $\epsilon$, $\sigma$ and *n* are scalar constants and ${\mathbf{r}}_{ij} = {\mathbf{r}}_{i} - {\mathbf{r}}_{j}$. Classical Lennard-Jones potential, with *n* specifically equals to 6, describes the potential energy between two interacting non-bonding particles. When two particles are close, they involve a steep increase of repulsive force; and when two particles are far away, there occurs a milder attractive force between each other. When the distance between two particles is equal to $\sigma$, the intermolecular potential is zero, and $\sigma$ is interpreted as the “soft diameter” of the particle.

Here, by integrating this “social distance” term into the crowd model, each individual can be interpreted as a soft sphere. Hence, $\sigma$ represents the imposed social distance when a social-distanced crowd is packed in a confined space. Moreover, *n* represents the “hardness” of spheres; that is, with a larger *n* value, the repulsive force will increase more sharply when two individuals get closer. In the context of the crowd model, *n* represents individuals’ priority to keep social distance in dynamic environments over other factors such as the desire of reaching a destination. As *n* increases, the individuals will be hypersensitive to social distance violations.

The “social force” between individual *i* and *j* is then given by

5 $$\begin{aligned} {\mathbf{F}}_{ij}({\mathbf{r}}_{ij}) = -c \nabla _{{\mathbf{r}}_{ij}} V_{ij}( \left\Vert {\mathbf{r}}_{ij}\right\Vert ) \end{aligned}$$

with *c* the direction dependent coefficient that accounts for the sight effect. When a neighbor is within the ± of $\theta$ away from the desired direction, the corresponding c is equal to 1. Otherwise, c can be a value smaller than 1. Considering the fact that, in crowds, attracting forces between individuals who are at large distances is not expected, we set for ${\mathbf{F}}_{ij}({\mathbf{r}}_{ij})$ to be zero if attracting forces occur. Removing the attracting forces makes the potential function piecewise, but this will not lead to numerical instability issues because there is no sharp jump at the transition point. From now on, we will call our generalized Lennard-Jones potential quasi-Lennard-Jones (quasi-L-J) potential.

In summary, the change of the velocity at any given time, for any given individual, is hence

6 $$\begin{aligned} \frac{d {\mathbf{v}}_i}{dt} = {\mathbf{F}}^{D}_i + \sum \limits _{j \ne i}^{} {\mathbf{F}}_{ij} + {\mathbf{F}}_{iW}. \end{aligned}$$

## Results

### Compare with traditional model

The advantage of our “social distance” term over the simple increasing of the repulsive range of any other potential function is that the fundamental physics is precisely described by the parameters (*n*, $\sigma$ and $\epsilon$) in the quasi-L-J potential. Thus, our “social distance” term offers the full flexibility of describing the different aspects of social-distanced crowds. By the definition of the quasi-L-J potential, $\sigma$ is the prescribed social distance. In addition, *n* describes the individual’s priority to keep the prescribed social distance. As *n* decreases, the social distance requirement is “softer”, and individuals tend to violate the social distance more often. Moreover, $\epsilon$ value couples with the other two acceleration terms to adjust the relative magnitude between the three acceleration terms in Eq. (6).

Traditional agent-based social force models^2,9,10^, which were designed without a prescribed social distance in mind, use an exponential function to describe the repulsive potential between individuals. One of the most popular potential function proposed by Helbing and Molnar^2^ is:

7 $$\begin{aligned} V_{ij}(b) = V_{ij}^{0}e^{-b/\delta }, \end{aligned}$$

where *b* is defined as the semiminor axis of equipotential line in the form of an ellipse that is dependent on the movement of the other individual *j* and is described as $b = \frac{1}{2}\times \sqrt{(\left\Vert {\mathbf{r}}_{ij}\right\Vert +\left\Vert {\mathbf{r}}_{ij}-v_{j}\Delta t \mathbf{e_j}\right\Vert )^2-(v_{j}\Delta t)^2}$, where $v_{j}\Delta t$ is of the order of the step width of individual *j* and $\Delta t$ is chosen to be 2 *s* in Helbing and Molnar^2^.

Even though this model also describes the increase of repulsive force as individuals approaching each other, the parameter setting lacks clear physical meaning in the context of social distance, hence makes the determination of parameter to be totally based on trial-and-error. Our new quasi-L-J potential, however, offers clear physical meaning for each parameter in the context of social distance.

**Figure 1:**
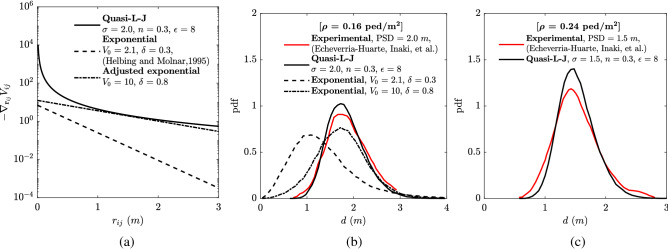
(**a**) The comparison of the negative gradient of potentials between individual *i* and *j* ($r_{i,j}$) for three different potential functions: quasi-L-J potential, Helbing and Molnar 1995 exponential potential^2^ and adjusted exponential potential. (**b**) The PDFs of *d* for three models with quasi-L-J potential, Helbing and Molnar 1995 exponential potential^2^, adjusted exponential potential, and the experimental PDF^12^ of *d*. (**c**) The comparison of the PDF of *d* from model with quasi-L-J potential and the experimental PDF^12^.

Figure 1a shows the negative gradient of potentials between individual *i* and *j* ($r_{i,j}$) for three different potential functions: quasi-L-J potential, Helbing and Molnar 1995 exponential potential^2^ and adjusted exponential potential. The determination of the parameters for the quasi-L-J potential is based on experimental data^12^. Since we observe that the Helbing and Molnar 1995 potential^2^ has repulsive force much smaller than the force from the quasi-L-J potentials at all distances, we adjust the two parameters of the exponential potential to make its negative gradient as close as possible to the one from the quasi-L-J potential and name the potential function as adjusted exponential potential. We observe that the two curves are very close when $r_{i,j}$ is relatively large (greater than 1 m in this case). For smaller $r_{i,j}$ (below 1 meter in this case), the quasi-L-J potential has a significant increase of the repulsive force than that from the adjusted exponential potential.

### Compare with experimental data

Partially due to the coronavirus pandemic, we could not conduct experiments to obtain suitable data on the social-distanced crowds^9,13,14^. We calibrate the model based on recent experimental work by Echeverrá-Huarte et al.^12^, which explores the dynamics of social-distanced walking pedestrians. We compare the resulting social distance PDFs from both our new model and the exponential models with the experimental social distance PDF to examine the performance of these models in reproducing the prescribed social distance between individuals in a social-distanced crowd. And we’ll show that, indeed, our model not only reproduces experimental data better but also have physically interpretable parameters which enable a more robust performance.

The work by Echeverrá-Huarte et al.^12^ measured the PDFs of the distance of each individual to the first nearest neighbor (*d*) under different prescribed safety distances (PSD) and crowd densities ($\rho$). Volunteers in this work were asked to walk randomly in an enclosed area, during which the movement of each individual was recorded and analyzed. To model this condition, we randomly assign individuals with different destinations on a circumscribed circle of a enclosed domain and alter the destinations randomly every k *s*. We find the model is robust for a relatively wide range of k and here we set k to be 4 *s*.

For a fair comparison between models, we keep ${\mathbf{F}}^{D}_i$ and ${\mathbf{F}}_{iW}$ terms same as that in Helbing and Molnar’s classical model^2^ for all models discussed here. Moreover, we keep the crowd density of our model the same as the crowd density in the experiments^12^ for comparisons with experimental data. We set $U_{iW} = 10$ m^2^ s^−2^ and $R = 0.2$ m. $\tau _i$ is set to be 0.5 *s*. The preferred speed $v_i^D$ and maximum speed $v_i^{max}$ of individuals are set to be 1.34 m s^−1^ and 1.74 m s^−1^. $c_w$ is chosen to be 1 in all cases while *c* is chosen to be 1 only if the neighbor individual is within $\theta = \pm 100^\circ$ of the focal individual’s desired direction (forward view); back view results in a *c* of 0.5. We conduct the modeling with 64 individuals in a 20 × 20 m^2^ closed room, which results in a crowd density of 0.16 ped/m^2^.

Considering the differences among individuals regarding their perceptions of social distance and their preferred walking speed, the $\sigma$ and $v_i^D$ are normally distributed among individuals with a mean of $\sigma$ and standard deviation 0.2$\sigma$ and a mean of $v_i^D$ and standard deviation 0.2$v_i^D$ respectively. The proper upper and lower limits are chosen to screen out unreasonable values for $\sigma$ and $v_i^D$. Also, to avoid gridlocks by balanced forces in symmetrical configurations, a small amount of stochastic irregularity is added. We simulate the crowd for 10 times with different initial random seeds for statistical robustness and convergence.

With this parameter setting, our quasi-L-J model achieved a PDF distribution of *d* very similar to that from experimental data^12^, where PSD = 2 m (Fig. 1b). Our model parameters are $\sigma$ = 2, *n* = 0.3 and $\epsilon$ = 8. We calibrated the model parameter *n* and $\epsilon$ to one significant number. The current discrepancy between the PDFs from quasi-L-J model and the experimental data will disappear if we keep more significant numbers for our model parameters.

By changing the parameter $V_0$ and $\delta$, the exponential potential can be adjusted to match our calibrated quasi-L-J potential when $r_{ij}$ is relatively larger, as is shown with the dot-dash line in Fig. 1a. However, one of the limitations of the exponential potential is that when $r_{ij}$ is small, it cannot achieve a sharper increase of repulsive force. In consequence, as is shown in Fig. 1b, the PDF value for smaller *d* with the adapted exponential potential has a long tail toward the smaller individual distance. This long tail of PDF toward smaller individual distances can result from the smaller repulsive forces between individuals at smaller distances.

Moreover, due to the lack of physical meaning for the parameters in the exponential potential in the context of social distance, the obtaining of the suitable parameter setting for the exponential potential relies on matching our calibrated quasi-L-J potential. Without the quasi-L-J model, the determination of the parameters for the exponential model would be totally based on trial-and-error in modeling social-distanced crowds. In addition, the traditional exponential potential needs to be totally re-calibrated if the crowd condition changed, such as the change of PSD. Since our quasi-L-J potential offers the full flexibility of describing different aspects of the social-distanced crowd, we do not need to re-calibrate our model if the crowd condition changed.

We have claimed that our model can prescribe social distance, and each parameter corresponds to different physics of the crowd, so our model should reproduce the crowd with a different social distance very well by only changing model parameter $\sigma$. Here, we test this idea with the experimental data^12^ with different PSD and $\rho$. Instead of re-calibrate our model, we simply change the our prescribed social distance ($\sigma$) and crowd density ($\rho$) in our modeling domain to the new conditions ($\sigma = 1.5$ m and $\rho = 0.24$ ped/m^2^). In Fig. 1c, we see that our potential can reproduce a PDF of *d* very similar to the experimental data in a different condition without the re-calibration.

## Discussions

**Figure 2:**
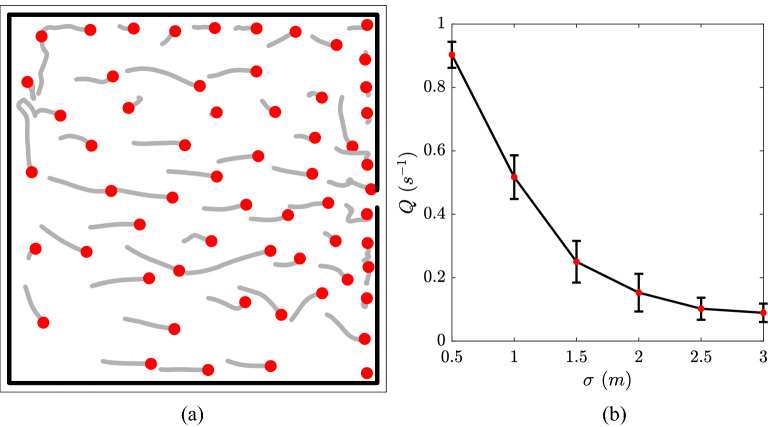
(**a**) Top view of the 20 m $\times$ 20 m simulation domain with trajectories during the 5 s previous to the the snapshot. (**b**) Flow rate *Q* out of the bottleneck with error bars as a function of mean social distance $\sigma$.

In order to demonstrate the usefulness of our model in enhancing transportation efficiency, we use our new calibrated model to tackle the bottleneck flow with a prescribed social distance. Bottlenecks are usually due to a narrow section of road, a junction, or a door that impedes pedestrian flow. To study the bottleneck motion base case, we specifically simulate people exiting a square room of 20 m by 20 m via a door located in the middle of the right wall, which is illustrated in Fig. 2a. The width of the door is chosen to be 36 in. (0.92 m), which is a common door width in the U.S. The simulation starts with 60 individuals with random initial locations, directions, and identical velocity magnitude of ${v_i^0}$. The destination of each individual is at a short distance outside of the door. Once an individual passes through the door, that individual is removed from the simulation. To keep the system at a statistically steady state, a periodic boundary condition is used, that is, once a individual is removed from the simulation, a new one would be randomly added near the vicinity of left wall with an uniformly distributed location between 0 and 20 m along the left wall. A sample of the trajectories during the 5 s previous to the snapshot is shown in Fig. 2a.

**Figure 3:**
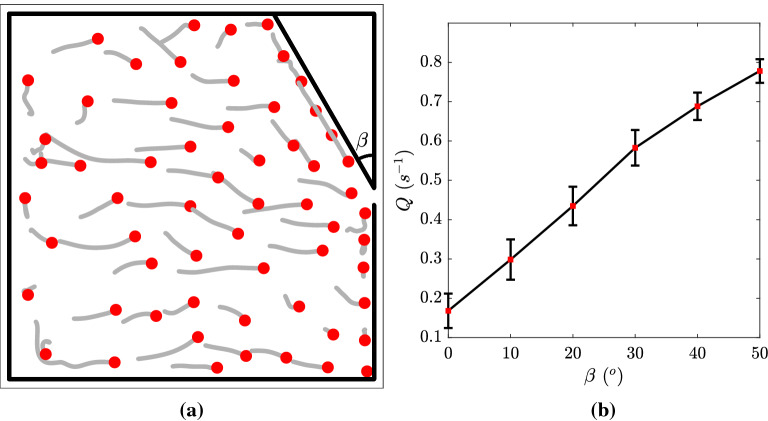
(**a**) Top view of the 20 m $\times$ 20 m simulation domain with imposed sidewall for introducing asymmetry. The angle between the sidewall and the original right wall is $\beta$. The gray lines are human trajectories during the 5 s previous to the the snapshot. (**b**) Flow rate *Q* out of the bottleneck as a function of $\beta$ with $\sigma = 2$ m and door width of 0.92 m.

**Figure 4:**
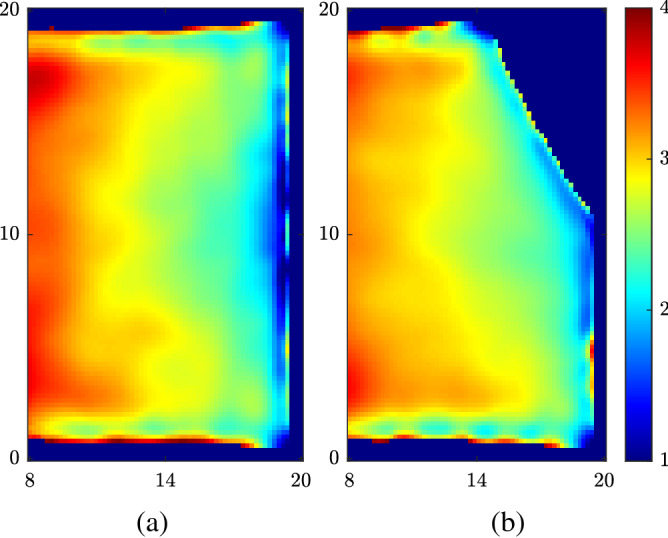
(**a**) Time-averaged distance between individuals without wall modification. (**b**) Time-averaged distance between individuals with a sidewall of $\beta = 30^\circ$. Length unit is in meters.

**Figure 5:**
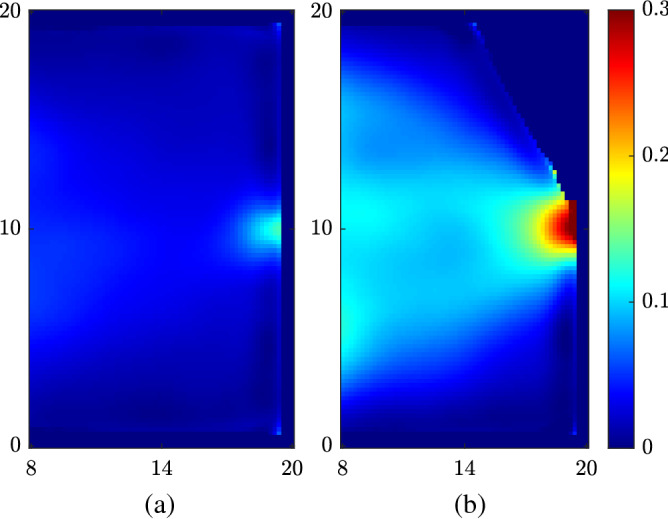
(**a**) Time-averaged kinetic energy ($\frac{1}{2}\left\Vert {\mathbf{v}}_i\right\Vert ^2$) of individuals without wall modification. (**b**) Time-averaged kinetic energy ($\frac{1}{2}\left\Vert {\mathbf{v}}_i\right\Vert ^2$) of individuals with a sidewall wall of $\beta = 30^\circ$. Length unit is in meters.

**Figure 6:**
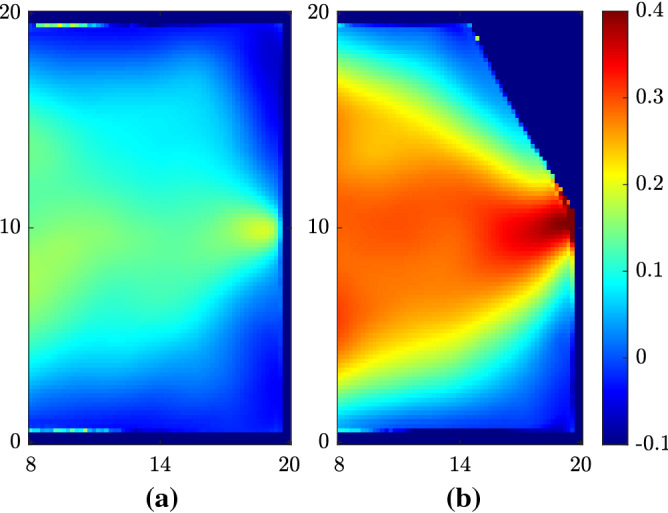
(**a**) Time-averaged efficiency (${\mathbf{v}}_i\cdot {\mathbf{e}}_i/\left\Vert {\mathbf{v}}_i^D\right\Vert$) of individuals without wall modification. (**b**) Time-averaged efficiency (${\mathbf{v}}_i\cdot {\mathbf{e}}_i/\left\Vert {\mathbf{v}}_i^D\right\Vert$) of individuals with a sidewall wall of $\beta = 30^\circ$.Length unit is in meters.

We first look into how different social distances impact the bottleneck effect. Since the social distance is usually larger than the door width, only one individual can pass through the door at a time and others have to wait beside the door. Another common condition is that when two or more individuals are trying to pass the same exit simultaneously, they can all get stuck near the door for few seconds in order to determine which one exits first. Therefore, a lower flow rate of people out of the door is expected with a larger social distance imposed. Indeed, as is shown in Fig. 2b, the flow rate *Q* (defined as the number of exited individuals per second) decreases significantly as the imposed social distance $\sigma$ increases beyond the door width. For each $\sigma$ value from 0.5 to 3 m, we run the simulation to calculate the flow rate *Q* after the simulation reaches a statistically steady state. The statistics of the simulation suggests that a larger social distance results in a much slower flow rate. Figure 4a shows the time-averaged spatial distribution of *d*. It is noticed that the individuals tend to have smaller social distances near the door because individuals may risk more to exit the room in the vicinity of the door.

A larger social distance considerably aggravates the bottleneck effect. Even though we can alleviate the bottleneck effect with a wider door, it is not practical to extensively modify the existing infrastructures. A natural question is: can small modifications of the existing bottlenecks increase Q?

With the assistance of this model, we find that a simple way to increase Q is to introduce asymmetry near the bottleneck. The intuition behind this strategy is simple. The bottleneck effect rises due to the narrow sections (such as doors), which all of the individuals try to get through. This causes the increase of the social forces between individuals that impede each other. Furthermore, on the group level, the net social force is not biased with respect to the center-line of the room through the exit (symmetry). So, statistically, group-level net social force is in balance, which intensifies the impedance of the movements and subsequently leads to a lower *Q*. In comparison, with asymmetry in boundary conditions, the net social force is biased in one direction and hence introduces a “mean flow” causing one side of the crowd to walk through the bottleneck with a higher possibility. This asymmetry leads to the break of a balanced group-level net “social force” and, thus, to an increased *Q*.

We introduce the asymmetry by adding a side wall near one side of the bottleneck that has a certain angle $\beta$ with the original right wall (Fig. 3a). Figure 3b shows the change of flow rate as a function of $\beta$. We can notice that the flow rate is significantly enhanced even by a small angle. One can notice that the flow rate with a 30° sidewall is more than three times the flow rate without a sidewall. This means that we do not need to sacrifice a great portion of the room space to achieve a considerable increase in flow rate. Another advantage of this method is its simplicity. We do not need to install actual walls in practice, but the changes in the boundary conditions could be done by simply adding some objects such as plants and shelves. We can get a further improvement of Q if we increase $\beta$ beyond 50°. However, the sidewall will block out a significant amount of area in the room and, thus, is not feasible. Additionally, as is shown in Fig. 4b, the modification of the boundary condition would not exacerbate the violation of social distancing requirement (*d* smaller than social distance). On the contrary, the violation is even alleviated near the bottleneck due to the asymmetry. Because of the asymmetry, one side of the individuals tend to exit with a much higher priority, and there is no close competition between individuals in the vicinity of the door.

Figures 5 and 6 further strengthen our explanation of the asymmetry mechanism. Figure 5 shows the time-averaged spatial distribution of the kinetic energy of individuals (defined as $\frac{1}{2}\left\Vert {\mathbf{v}}_i\right\Vert ^2$) and Fig. 6 shows the time-averaged spatial distribution of the efficiency of individuals (defined as ${\mathbf{v}}_i\cdot {\mathbf{e}}_i/\left\Vert {\mathbf{v}}_i^D\right\Vert$)^9^. Both symmetrical and asymmetrical conditions are simulated with $\sigma = 2$ m and door width of 0.92 m. $\beta$ is chosen to be $30^\circ$. It is obvious that both the kinetic energy and efficiency of individuals show an asymmetrical distribution with the sidewall, which indicates that the sidewall indeed increases the transportation efficiency Q by breaking the symmetry. Moreover, the absolute magnitudes of these two parameters are also significantly increased with the sidewall, which is consistent with the result of Fig. 3b. Specifically, the sidewall increases the asymmetry of efficiency more than that of the kinetic energy. This suggests that the sidewall increase the transportation efficiency Q by facilitating individuals to walk in their desired directions than by prominently increasing individuals’ speeds.

Our result demonstrates that the low efficiency due to the extra “buffer” distance between individuals can be significantly increased by adding a small modification: a sidewall. The added sidewall increases transportation efficiency by introducing asymmetry. Even though it looks similar to the obstacle effect, these two cases are intrinsically different. The term “obstacle effect” describes the observation that under emergency evacuation conditions, the transportation at the bottleneck can be counterintuitively increased by a suitably placed obstacle near the exit. Many researchers have explored this topic using both simulation^15,16^ and empirical^17,18^ methods regarding, to name a few, the effect of obstacle size and position^19,20^, and the effect of corner exit rather than middle exit^21,22^. However, the obstacle and corner exit effects are applied to emergency escape conditions where the clogging results from the extremely small distance between individuals. There might involve direct contact and even pushing between individuals. Kirchner et al.^15^ showed that the introduction of friction between pedestrians is essential for significant obstacle effect. Helbing et al.^17^ showed that without pushy behavior, where the pressure exceeds a certain level, the outflow rates for rooms with and without exit obstacles were about the same. On the contrary, under the social-distanced condition, the clogging is a consequence of the large social distance, which is intrinsically different from the emergency escape condition. It could be interesting to test whether such effects also exist for a crowd with large social distance, but it is out of the scope of this paper.

## Conclusions

We introduced a “social distance” term and integrated it into a widely accepted agent-based social force model to describe the movement of crowds under social-distanced conditions during the pandemic and post-pandemic periods. The advantage of this “social distance” term over the simple increasing of the repulsive range of other alternatives is that the fundamental crowd physics is precisely described by the model parameters offering the full flexibility in describing the different aspects of the social-distanced crowds. We compared our model with the classical model that uses an exponential repulsive potential between individuals rather than our new quasi-L-J potential. Then, we tested the resulting social distance PDFs from both models against experimental results. We found that our model performs better in reproducing experimental results and is more robust with the change of crowd conditions. To demonstrate the usability of our model, we focused on the bottleneck case and showed that the bottleneck flow rate can be significantly increased by some small modifications. We demonstrated only one of the possibilities to enhance transportation through the bottleneck, that is, adding a side wall near the bottleneck, but there is a wide range of research possibilities with this new social force model. Moreover, many more motion base cases in pandemic and post-pandemic period can be studied with this model^10^.

## References

[1] Helbing, D. & Johansson, A. Pedestrian, crowd, and evacuation dynamics. arXiv preprint arXiv:1309.1609 (2013).

[2] Helbing D, Molnar P (1995). Social force model for pedestrian dynamics. Phys. Rev. E.

[3] Helbing D, Farkas IJ, Vicsek T (2000). Freezing by heating in a driven mesoscopic system. Phys. Rev. Lett..

[4] Ouellette NT (2019). Flowing crowds. Science.

[5] Treiber, M. Crowd flow modeling of athletes in mass sports events: A macroscopic approach, in *Traffic and Granular Flow’13*, 21–29 (Springer, 2015).

[6] Löhner R (2010). On the modeling of pedestrian motion. Appl. Math. Model..

[7] Kang, W. & Han, Y. A simple and realistic pedestrian model for crowd simulation and application. arXiv preprint arXiv:1708.03080 (2017).

[8] Bain N, Bartolo D (2019). Dynamic response and hydrodynamics of polarized crowds. Science.

[9] Helbing D, Farkas I, Vicsek T (2000). Simulating dynamical features of escape panic. Nature.

[10] Chen X, Treiber M, Kanagaraj V, Li H (2018). Social force models for pedestrian traffic-state of the art. Transp. Rev..

[11] Jones JE (1924). On the determination of molecular fields.—II. From the equation of state of a gas. Proc. R. Soc. Lond. Ser. A.

[12] Echeverría-Huarte I, Garcimartín A, Hidalgo R, Martín-Gómez C, Zuriguel I (2021). Estimating density limits for walking pedestrians keeping a safe interpersonal distancing. Sci. Rep..

[13] Kretz T, Grünebohm A, Schreckenberg M (2006). Experimental study of pedestrian flow through a bottleneck. J. Stat. Mech: Theory Exp..

[14] Hoogendoorn SP, Daamen W (2005). Pedestrian behavior at bottlenecks.. Transp. Sci..

[15] Kirchner A, Nishinari K, Schadschneider A (2003). Friction effects and clogging in a cellular automaton model for pedestrian dynamics. Phys. Rev. E.

[16] Echeverría-Huarte I, Zuriguel I, Hidalgo R (2020). Pedestrian evacuation simulation in the presence of an obstacle using self-propelled spherocylinders. Phys. Rev. E.

[17] Helbing D, Buzna L, Johansson A, Werner T (2005). Self-organized pedestrian crowd dynamics: Experiments, simulations, and design solutions. Transp. Sci..

[18] Feliciani C, Zuriguel I, Garcimartín A, Maza D, Nishinari K (2020). Systematic experimental investigation of the obstacle effect during non-competitive and extremely competitive evacuations. Sci. Rep..

[19] Shi X, Ye Z, Shiwakoti N, Grembek O (2018). A state-of-the-art review on empirical data collection for external governed pedestrians complex movement. J. Adv. Transp..

[20] Zuriguel I (2016). Effect of obstacle position in the flow of sheep through a narrow door. Phys. Rev. E.

[21] Shi X, Ye Z, Shiwakoti N, Tang D, Lin J (2019). Examining effect of architectural adjustment on pedestrian crowd flow at bottleneck. Physica A.

[22] Liu, Y., Shi, X., Ye, Z., Shiwakoti, N. & Lin, J. Controlled experiments to examine different exit designs on crowd evacuation dynamics, in *CICTP 2016*, 779–790 (2016).

